# Lack of common TCRA and TCRB clonotypes in CD8^+^/TCRαβ^+^ T-cell large granular lymphocyte leukemia: a review on the role of antigenic selection in the immunopathogenesis of CD8^+^ T-LGL

**DOI:** 10.1038/bcj.2013.70

**Published:** 2014-01-10

**Authors:** Y Sandberg, M J Kallemeijn, W A Dik, D Tielemans, I L M Wolvers-Tettero, E J van Gastel-Mol, T Szczepanski, Y Pol, N Darzentas, J J M van Dongen, A W Langerak

**Affiliations:** 1 Department of Immunology, Erasmus MC, University Medical Center Rotterdam, Rotterdam, The Netherlands; 2 Department of Pediatric Hematology and Oncology, Medical University of Silesia, Zabrze, Poland; 3 Institute of Agrobiotechnology, Center for Research and Technology Hellas, Thessaloniki, Greece

**Keywords:** T-LGL, TCRB, TCRA, CDR3, HLA, antigenic stimulation

## Abstract

Clonal CD8^+^/T-cell receptor (TCR)αβ^+^ T-cell large granular lymphocyte (T-LGL) proliferations constitute the most common subtype of T-LGL leukemia. Although the etiology of T-LGL leukemia is largely unknown, it has been hypothesized that chronic antigenic stimulation contributes to the pathogenesis of this disorder. In the present study, we explored the association between expanded TCR-Vβ and TCR-Vα clonotypes in a cohort of 26 CD8^+^/TCRαβ^+^ T-LGL leukemia patients, in conjunction with the HLA-ABC genotype, to find indications for common antigenic stimuli. In addition, we applied purpose-built sophisticated computational tools for an in-depth evaluation of clustering of TCRβ (TCRB) complementarity determining region 3 (CDR3) amino-acid LGL clonotypes. We observed a lack of clear TCRA and TCRB CDR3 homology in CD8^+^/TCRαβ^+^ T-LGL, with only low level similarity between small numbers of cases. This is in strong contrast to the homology that is seen in CD4^+^/TCRαβ^+^ T-LGL and TCRγδ^+^ T-LGL and thus underlines the idea that the LGL types have different etiopathogenesis. The heterogeneity of clonal CD8^+^/TCRαβ^+^ T-LGL proliferations might in fact suggest that multiple pathogens or autoantigens are involved.

## Introduction

Large granular lymphocyte (LGL) proliferations are derived from normal cytotoxic LGL cells, which comprise 10–15% of peripheral blood (PB) mononuclear cells (MNCs).^1, 2, 3^ The majority of the normal LGL cells (85%) are of NK-cell origin, and a minority is derived from mature (post-thymic) T lymphocytes. Lymphoproliferations of LGLs range from activated polyclonal expansions to clinically overt leukemias. T-cell LGL (T-LGL) leukemia is the most common subtype, representing ∼85% of all LGL leukemia cases diagnosed in western countries.

T-LGL leukemia is a rare and heterogeneous disorder and about one-third of patients is asymptomatic at diagnosis. The main clinical manifestations are related to chronic neutropenia and/or anemia. ^3, 4, 5, 6, 7^ There is a frequent association with a wide variety of autoimmune diseases (33%) and other malignancies (13%).^8^ Its diagnosis is based on a persistent (>6 months) morphologically and/or immunophenotypically increased clonal CD3^+^/CD57^+^ LGL population in PB, usually >2 × 10^9^/l, though a lower count (range 0.4–2 × 10^9^/l) may also be compatible with a diagnosis of T-LGL leukemia.^9, 10^ T-LGL leukemias can be divided into three groups on the basis of their immunophenotypical and molecular characteristics: CD8^+^, CD4^+^ and T-cell receptor (TCR)γδ^+^ T-LGL. Monoclonal CD8^+^/TCRαβ^+^ T-LGL leukemia forms the largest subgroup (80–90%) of monoclonal T-LGL lymphoproliferative disorders.^11^ It presents in elderly individuals (mean age 60 years) and generally has an indolent clinical course.^3, 12^ However, cases with a more aggressive clinical course that are associated with a CD3^+^/CD8^+^/CD56^+^/CD57^−^ phenotype have been reported as well.^13, 14^ CD3^+^/CD4^+^/TCRαβ^+^ T-LGL leukemia and CD3^+^/TCRγδ^+^ T-LGL leukemia are far less common (<5 and 5–10%, respectively), but a considerable number of cases from both disease entities have recently been described in detail.^15, 16^

Clonality assessment via PCR-based studies of TCR genes is essential to discriminate true T-LGL leukemia from other reactive proliferations. It should be stressed that the finding of clonality does not necessarily imply malignancy in this disease, since most cases are indolent and do not require therapy. Therefore, patients are often diagnosed as having T-cell clonopathy of undetermined significance.^1, 4, 17^

Although the etiology of T-LGL leukemia is still largely unknown, it has been hypothesized that chronic antigenic stimulation contributes to the pathogenesis of this disorder. This is in line with the activation-associated effector phenotype and the skewed TCR expression pattern found in T-LGL leukemia. An exaggerated response to immunodominant autoantigens or viral/bacterial antigens might be the initial step in the development of this disorder.^18, 19, 20, 21^ Recently, the T-cell repertoire has been demonstrated to be dynamic in a large proportion (37%) of T-LGL leukemia patients, a phenomenon referred to as ‘clonal drift'.^22^ This supports the hypothesis that extreme clonal evolution is the result of a polarized reactive process. On top of that, secondary molecular events are assumed to be required to establish the full leukemic phenotype of the chronically antigen-stimulated T-LGL population. Those events especially lead to dysregulated apoptosis and constitutively activation of multiple cell survival pathways. ^11, 12, 23, 24, 25, 26^

To further substantiate the potential involvement of a common antigen in driving development of clonal T-LGL proliferations, the complementarity determining region 3 (CDR3) sequences of the rearranged TCR genes are being analyzed. The CDR3 region of the TCR molecule has the highest antigenic specificity and directly binds to the antigenic peptide presented in the context of HLA.^27^ Garrido *et al.*^28^ demonstrated strikingly similar motifs in CDR3 TCR-Vβ13 sequences in 42% of CD4^+^/TCRαβ^+^ T-LGL leukemia cases and a clear association with the HLA-DR*0701 genotype. Interestingly, highly similar CDR3 sequences could also be detected in TCRγ (TCRG) and TCRδ (TCRD) genes in nearly half of patients diagnosed with TCRγδ^+^ T-LGL leukemia, supporting a common antigen-driven origin of this disorder.^16^

In CD8^+^/TCRαβ^+^ T-LGL leukemia non-random clonal selection has been suggested,^18, 29^ even though no consistent single structural homologous motif could be detected in CDR3 sequences of TCRβ (TCRB) genes, The seeming lack of such identical TCR specificities could however also be explained by the diverse HLA background of these patients. Furthermore, the TCRα chain might have an important role next to the TCRβ chain, especially in the initial phase of high-affinity clonal TCR selection.^30^ However, the CDR3 regions of the TCRα (TCRA) genes have not been extensively studied in CD8^+^/TCRαβ^+^ T-LGL leukemia.

In the present study, we therefore explored the existence of a potential association between CDR3 sequences of both the TCRA and TCRB clonotypes in a cohort of 26 patients diagnosed with CD8^+^/TCRαβ^+^ T-LGL leukemia in conjunction with the HLA genotype. In addition, we applied purpose-built sophisticated computational tools, specifically developed for sequence pattern discovery in CDR3 amino-acid sequences to evaluate clustering of a large number of TCRB CDR3 amino-acid clonotypes of CD8^+^/TCRαβ^+^ T-LGL leukemia patients.

## Materials and methods

### Patients and cell samples

PB and/or bone marrow (BM) samples from 26 patients with CD8^+^/TCRαβ^+^ T-LGL leukemia were obtained. The diagnosis of T-LGL leukemia was established by clinical and laboratory parameters as defined previously.^7, 9, 31^ Patients with a persistent (>6 months) and increased (>1 × 10^9^/l) monoclonal CD3^+^/CD8^+^/TCRαβ^+^ T-LGL proliferation in PB were included. All patient samples were obtained according to the Helsinki declaration following guidelines of the Medical Ethics Committee of Erasmus MC, University Medical Center (Rotterdam, The Netherlands). PB/BM MNCs were isolated by Ficoll-Paque (density: 1.077 g/ml; Pharmacia, Uppsala, Sweden) centrifugation and were used for DNA isolation and RNA isolation. Immunophenotyping was performed on whole PB or BM samples and occasionally on MNC fractions. Cytomorphological May-Grünwald-Giemsa staining of PB smears was used for morphological evaluation of LGLs. HLA genotyping for HLA-ABC was performed by Luminex-based SSOP-PCR techniques (One Lambda Inc., Canoga Park, CA, USA).

### Immunophenotypical analysis

Cells were analyzed for membrane expression using a routine panel of monoclonal antibodies, including CD2, CD3, CD4, CD5, CD7, CD8, CD16, CD56, CD57, anti-TCRαβ (BMA031 and WT31), anti-TCRγδ (11F2) and anti-HLA-DR. Immunofluorescence stainings were performed as described^32^ and evaluated on a FACSCalibur or FACSCanto II (BD Biosciences, San Jose, CA, USA) flow cytometer. Data analysis was performed using CellQuest and Paint-A-Gate Pro software (BD Biosciences).

The PB and/or BM samples were studied in more detail for Vβ domain expression to quantify the contribution of each Vβ family to the CD8^+^ lymphocyte population. To this end, flow-cytometric analysis was performed using the IO Test Beta Mark kit (Beckman Coulter, Brea, CA, USA) as described.^32^ Samples in which the Vβ restriction of the expansion could not be identified by flow cytometry were analyzed by TCRB RT–PCR as described.^33^

### DNA and RNA isolation and cDNA synthesis

High-molecular weight DNA from fresh or frozen PB MNCs was extracted using a phenol-chloroform extraction-based protocol, followed by ethanol precipitation and resolution in Tris-EDTA buffer.^34^ In a subset of cases, DNA was isolated using the GenElute Mammalian Genomic DNA miniprep kit (Sigma-Aldrich, St Louis, MO, USA) according to the manufacturer's protocol. Total RNA was extracted from fresh or frozen PB and/or BM MNCs from patients and reverse transcribed into cDNA as previously described.^35^ cDNA quality was checked using *ABL* as a control gene.

### TCRA and TCRB gene rearrangement analysis

For TCRA gene rearrangement analysis, cDNA was amplified using newly developed TCRA primers: one constant region reverse primer (Cα) and 54 different Vα family-specific forward primers distributed over 5 different multiplex tubes; each of these multiplex contained 10 (TCRA tube B) or 11 (TCRA tubes A, C, D and E) Vα primers in combination with the Cα primer (Supplementary Table 1). In each 50 μl PCR, 2 μl of cDNA, 10 pmol of 5′ and 3′ oligonucleotide primers, 3 mmol/l MgCl_2_, 0.2 mmol/l dNTP, 5 μl 10 × buffer II, and 1–2 U Ampli*Taq* Gold polymerase (Applied Biosystems, Foster City, CA, USA) were used.

TCRB gene rearrangement analysis was performed according to the BIOMED-2 multiplex PCR protocol.^36^ BIOMED-2 multiplex PCR kits were obtained from InVivoScribe Technologies (San Diego, CA, USA; http://www.invivoscribe.com). Amplification reactions were performed in an automated thermocycler (model ABI 2700; Applied Biosystems).

### Sequence analysis

After PCR amplification of TCRA and TCRB gene rearrangements, products were subjected to heteroduplex analysis.^37^ Products found to be monoclonal in heteroduplex analysis were directly sequenced except for cases with more than one clonal product. In such cases, homoduplexes were excised from the polyacrylamide gel and DNA was eluted before sequencing. Sequencing was performed on the ABI 3100 or 3130xl Genetic Analyzers (Applied Biosystems), using the dye terminator cycle sequencing kit and Ampli*Taq*FS DNA polymerase (Applied Biosystems). Assignment of Vβ, Dβ, Jβ, Vα and Jα genes and reading frames of the involved TCRB and TCRA gene rearrangements was done using the IMGT database (http://www.imgt.org).^38^

### *In silico* analysis and data visualization

V-J combinatorial diversity was visualized using Circoletto, an online visualization tool based on Circos (http://bat.ina.certh.gr/tools/circoletto/).^39^

The collected TCRB CDR3 amino-acid diversity was further analyzed using the TEIRESIAS algorithm, a computational tool developed by the Bioinformatics and Pattern Discovery group at the IBM Computational Biology Center, as described previously.^40^ This algorithm uses a motif-based clustering approach with predefined thresholds for amino-acid identity and similarity, CDR3 length differences and offsets for sequence motifs within CDR3 sequences.

TCRB CDR3 amino-acid patterns of different subsets were visualized using Weblogo (http://weblogo.berkeley.edu/). Each logo consists of multiple stacks of symbols, one stack for each position of the sequence. CDR3 is shown based on IMGT position definitions.

## Results

### Clinical and hematological features are heterogeneous in CD8^+^/TCRαβ^+^ T-LGL leukemia

The most relevant clinical and hematological findings at diagnosis of the 26 CD8^+^/TCRαβ^+^ T-LGL leukemia patients enrolled in this study are summarized in Table 1. The median age was 58 years (range 31–86 years) and there was no male or female predominance. Out of the 26 patients, 21 (81%) were symptomatic at presentation. Nine of the twenty-six patients (35%) had an episode of bacterial infection or B symptoms (fever, night sweats and weight loss). Most frequent presentations concerned neutropenia and/or anemia (62%), whereas some T-LGL leukemias presented with neutropenia plus thrombocytopenia (12%). Thrombocytopenia with coexistent anemia was found in just one case (4%), splenomegaly in two (8%) and lymphadenopathy also in only one (4%). Examination of PB smears showed an increased number of LGLs with abundant cytoplasm containing azurophilic granules in virtually all analyzed cases.

An associated disease was found in 11 cases (42%) (Table 1). In our cohort, seven patients (27%) had a co-existent autoimmune disorder. The most common autoimmune manifestation was rheumatoid arthritis, which was diagnosed in three patients (12%). Co-existence of a malignancy was found in five cases (18%), three of which showed a second hematological malignancy (12%).

**Figure 1:**
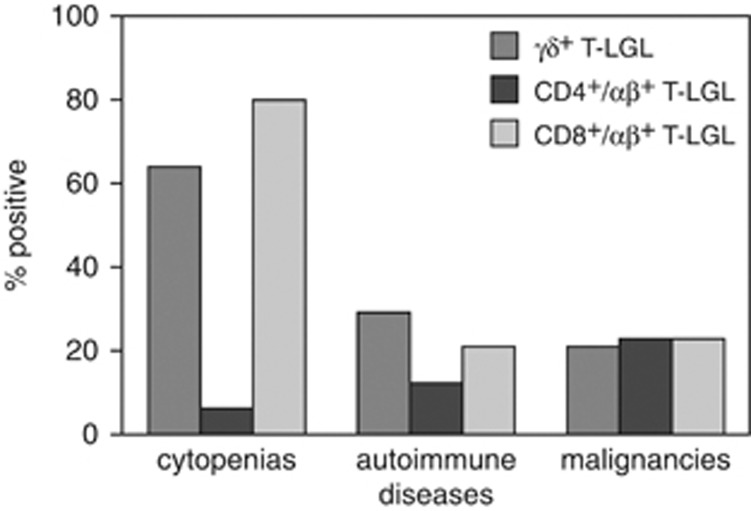
Relative frequency (%) of associated cytopenias, autoimmune diseases and malignancies in CD8^+^/TCRαβ^+^, CD4^+^/TCRαβ^+^ and TCRγδ^+^ T-LGL leukemia.

To compare the relative frequencies of associated cytopenias, autoimmune disorders and malignancies in all three types of T-LGL leukemia, we analyzed the clinical data of our CD8^+^ T-LGL cohort (*n*=26) and the data of 56 CD8^+^ T-LGL leukemia patients described by Wlodarski *et al.*^29^ and compared those with the clinical features of 36 patients with CD4^+^ T-LGL leukemia^28^ and our cohort of 44 published^16^ and 19 novel TCRγδ^+^ T-LGL leukemia patients. On average, similar clinical features were observed between CD8^+^/TCRαβ^+^ T-LGL leukemia and TCRγδ^+^ T-LGL leukemia. Notably, the frequency of cytopenias and autoimmune disorders appeared to be much lower in CD4^+^/TCRαβ^+^ T-LGL leukemia as compared with other types (Figure 1).

At closing of the study, the median follow-up of the patients was 34 months (range 6–122 months). Two-thirds of patients required therapy with one or more agents (Table 1). The therapeutic strategy was largely aimed at improving cytopenias and included erythrocyte transfusions and various immunosuppressive drugs. We observed one disease-related death in these 26 patients (case 86-041) which is in line with the generally indolent clinical course of this disease.

### Typical LGL immunophenotype is seen in all CD8^+^/TCRαβ^+^ T-LGL

T-LGL cells of all 26 cases showed membrane co-expression of CD3, CD8 and TCRαβ molecules. The majority of leukemic LGLs expressed CD2 (100%), CD5 (77%) and CD7 (81%). Furthermore, the T-LGL cells of all cases showed expression of one or more markers like CD16, CD56 or CD57 that have typically been associated with LGL and that reflect the antigen-experienced nature of the cells (Table 1). Out of 22 cases 11 were CD16 positive (50%), whereas CD56 expression was seen in 7 out of 20 cases analyzed (35%). Heterogeneous expression of CD57 could be demonstrated in 23 out of 24 evaluable cases (96%). Thus, these CD8^+^/TCRαβ^+^ T-LGLs show the typical effector T-cell phenotype.

### TCRA and TCRB combinatorial diversity greatly differs between CD8^+^/TCRαβ^+^ T-LGL and CD4^+^/TCRαβ^+^ T-LGL

To evaluate whether CD8^+^/TCRαβ^+^ T-LGL shows signs of antigen stimulation in their antigen receptors, we aimed for a comprehensive analysis of both the TCRα and the TCRβ chain in parallel to the HLA genotype.

First, we studied the clonotypic TCRB repertoire using specific anti-TCR Vβ domain MoAbs. Dominant TCR-Vβ reactivity was observed in 22 out of 26 cases (Table 2). All cases, including the four cases without detectable TCR Vβ expression, demonstrated clonal in-frame TCRB gene rearrangements in multiplex PCR and/or RT–PCR analysis (Table 3). Results of sequence analysis of Vβ-Jβ gene rearrangements and Vβ protein/mRNA expression were concordant in all cases. On the basis of these results, a slight predominance of Vβ2, Vβ5 and Vβ12 was noted. Jβ2 genes were used more frequently than Jβ1 genes (62 vs 38%), with the Jβ2.1 proportion being highest (15%). This Jβ2 predominance is in line with the non-random Jβ gene distribution as it is known from mature polyclonal PB TCRαβ^+^ T cells of healthy individuals.^41^

To study the clonotypic TCRA repertoire, we developed a novel multiplex RT-PCR assay. Using this assay, clonal TCRA gene rearrangements could be demonstrated in all 22 cases that were analyzed (Table 2). In the remaining four cases no RNA could be isolated due to lack of material. Similar to Vβ usage, there was also no common Vα gene usage among the 22 patients studied. Genes from the Vα19, Vα8 and Vα12 families were used most frequently, being expressed in three cases each. Jα gene usage was also highly diverse (Table 4).

**Figure 2:**
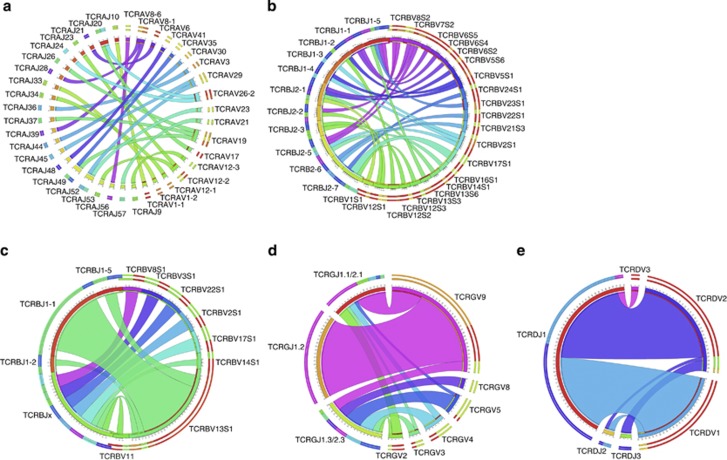
Frequencies of V-J pairing in TCRαβ^+^ T-LGL and TCRγδ^+^ T-LGL leukemia. Highly diverse Vα-Jα (**a**) and Vβ-Jβ (**b**) combinations are seen in our cohort of 26 patients with CD8^+^ T-LGL leukemia, while the Vβ-Jβ pairing is clearly non-random in monoclonal CD4^+^ T-LGL proliferations (**c**). Also in TCRγδ^+^ T-LGL leukemia limited combinatorial diversity of TCRG and TCR genes (**d** and **e**, respectively) is seen. (Blue to purple rectangular bands) J genes and (red to cyan rectangular bands) V genes. The width of the bands is proportional to the number of times the V and J genes are connected. This figure was generated using the Circos software package.^47^

When visualizing the TCRA and TCRB combinatorial diversity using the Circoletto tool, both the Vα-Jα and Vβ-Jβ diversities were indeed largely random in CD8^+^/TCRαβ^+^ T-LGL (Figures 2a and b). This is in strong contrast to the non-random distribution of Vβ-Jβ combinations as seen in monoclonal CD4^+^/TCRαβ^+^ T-LGL lymphocytosis patients.^28^ (Figure 2c); unfortunately, from these CD4^+^/TCRαβ^+^ T-LGLs no Vα-Jα combinatorial data are available for a direct comparison of the TCRA combinatorial diversity. For a complete picture on the combinatorial diversity of all three T-LGL subtypes, we also analyzed the Vγ-Jγ and Vδ-Jδ combinations in our cohort of 63 TCRγδ^+^ LGL patients using Circoletto. Similar to the diversity in CD4^+^/TCRαβ^+^T-LGL, but unlike CD8^+^/TCRαβ^+^ T-LGL, the combinatorial diversity in TCRG and TCRD appeared to be non-random as well (Figures 2d and e). It should be noted that the lower number of V genes might already impact on the more limited combinatorial diversity of these two loci.

Finally, we evaluated the HLA-ABC genotype of the CD8^+^/TCRαβ^+^ T-LGL patients. No clear predominance of HLA-A, -B or -C alleles or combinations thereof was observed (Table 2). When evaluating the expanded Vβ and Vα families in conjunction with the HLA-ABC alleles, also no clear association was observed between a particular TCR-Vα/Vβ specificity and the involved HLA genotype.

Collectively, these data show a clear heterogeneity in the combinatorial diversity of the TCR clonotypes in CD8^+^/TCRαβ^+^ T-LGL, which does not seem to be directly linked to the HLA genotype. This heterogeneity clearly differs from the more homogeneous patterns seen in TCRγδ^+^ T-LGL and especially CD4^+^/TCRαβ^+^ T-LGL.

### Lack of common TCRA and TCRB CDR3 motifs in CD8^+^/TCRαβ^+^ T-LGL

Given the lack of skewing of TCRA and TCRB combinatorial diversity, we then explored the possibility of a more subtle TCR homology in CD8^+^/TCRαβ^+^ T-LGL leukemia patients. To this end, we analyzed TCRA and TCRB junctional diversity by studying CDR3 sequences in more detail. A total of 24 T-LGL TCRA CDR3 clonotypes from 22 patients and 27 T-LGL TCRB CDR3 clonotypes from 25 patients were evaluated, but the TCRA and TCRB CDR3 motifs of the immunodominant T-cell clones did not show clearly identical sequences (Tables 3 and 4).

To exclude the possibility that the number of evaluable TCRB CDR3 sequences was limiting the possibility to find clear similarities, additional CDR3 sequences that had been published in the literature were included for further analysis. In this way, 81 additional TCRB CDR3 motifs of a large series of 56 CD8^+^/TCRαβ^+^ T-LGL leukemia patients^29^ could be evaluated. Similar to our cohort, in a proportion of patients more than one immunodominant clone was found, suggesting that some T-LGL proliferations are biclonal. In this extended data set, the Vβ-Jβ combinatorial diversity appeared to be equally heterogeneous (Supplementary Figure 1) as in our own cohort.

**Figure 3:**
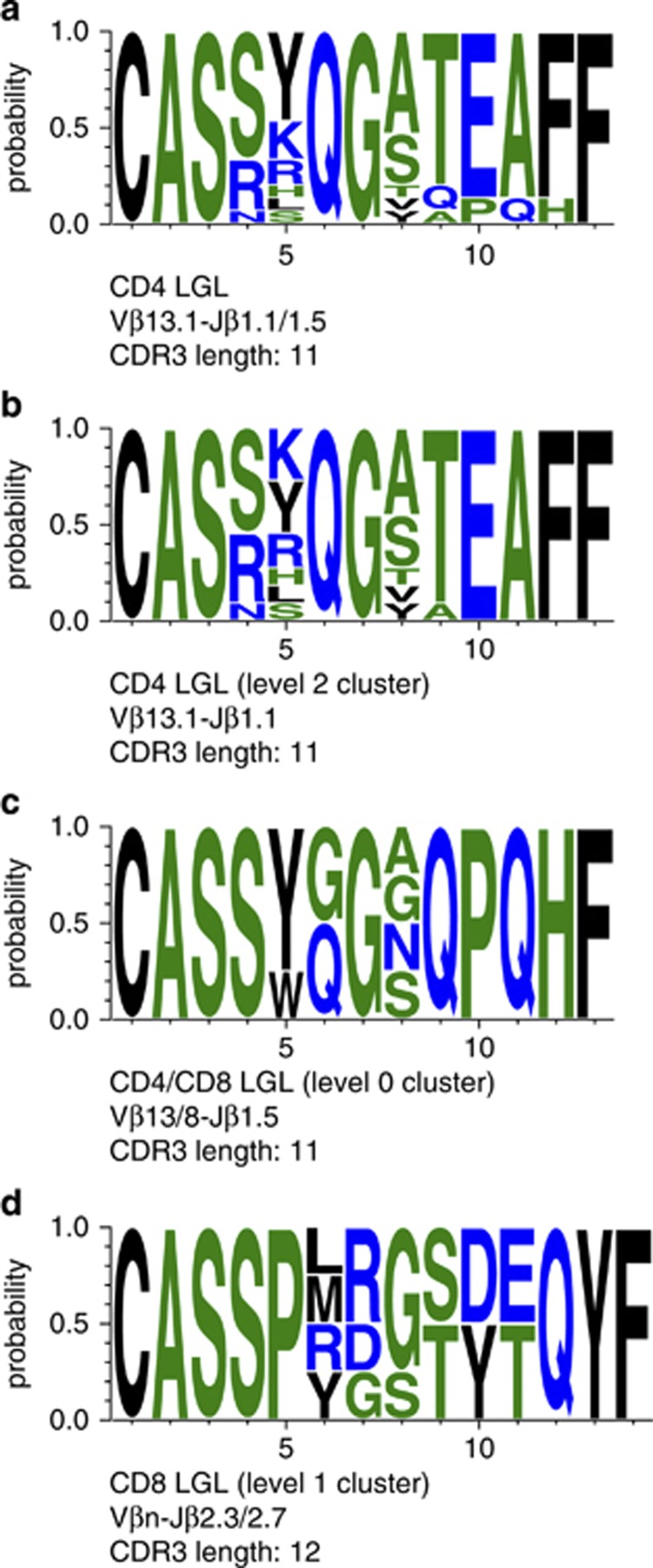
Sequence logos of selected subsets in T-LGL leukemia. (**a**) Subset 1 comprises 13 Vβ13.1-Jβ1.1/ Jβ1.5 gene rearrangements in CD4^+^ T-LGL leukemia cases, characterized by pronounced overall similarity. (**b**) The largest high-level subset in the present study is present in subset 2, comprising clonally expanded CD4^+^ T-LGL showing Vβ13.1-Jβ1.1. (**c**) Low level clustering was seen in Vβ8/Vβ13-Jβ1.5 gene rearrangements in two CD4^+^ LGL and two CD8^+^ LGL cases. (**d**) Some higher level clustering is present in Vβn.-Jβ2.3/2.7 gene rearrangements in four CD8^+^ T-LGL leukemia cases. The height of symbols within the stack indicates the relative frequency of each amino acid at that position. Amino-acid position is according to the IMGT numbering for the V domain. This figure was generated using WebLogo (http://weblogo.berkeley.edu/).

For a more comprehensive evaluation of CDR3 motifs, detailed *in silico* analysis was performed on the 108 combined TCRB CDR3 sequences in parallel to 14 TCRB CDR3 sequences of earlier described CD4^+^ T-LGL.^28^ By applying a recently described sequence motif-based clustering methodology^40^ using thresholds of 50% amino-acid identity and 70% similarity between any two CDR3 sequences, 13 out of 14 CD4^+^ T-LGL displayed a highly homogeneous and similar TCR with clear similarities in length and amino-acid positions in the CDR3 sequence logo (Figure 3a). The similarity of the CDR3 sequence logo was even more impressive when concentrating on a higher level cluster of 11 CD4^+^ T-LGL cases that are all characterized by TCRVβ13.1-Jβ1.1 rearrangements (Figure 3b). This is in line with the proposed CMV antigen-driven selection in the pathogenesis of this type of T-LGL. Interestingly, the two CD4^+^ T-LGL that were slightly different from the other 11 CD4^+^ T-LGLs based on Jβ1.5 usage did show low level clustering with two CD8^+^ T-LGLs, as evidenced from the common Jβ1.5 and Vβ8/Vβ13 gene usage, and the CDR3 sequence logo (Figure 3c). Finally, some clustering was seen between four other CD8^+^ T-LGL cases that were characterized by Jβ2.3/2.7 usage and a CDR3 length of 12 (Figure 3d). Unfortunately not enough TCRA sequences were available for a meaningful *in silico* TCRA CDR3 analysis.

Collectively the *in silico* analyses illustrate that, in strong contrast to CD4^+^ T-LGL, CD8^+^ T-LGL do not show clear and consistent signs of TCR homology that would reflect involvement of a common antigen.

## Discussion

Molecular analysis of the TCR repertoire can be a powerful tool in the study of T-cell responses to pathogens and in autoimmune diseases. Thus, analysis of the TCR expression pattern in patients with T-LGL leukemia might provide insight into the pathogenesis of this disorder. Similar TCR clonotypes between T-LGL clones of different patients would in that respect be suggestive of a common antigenic stimulus underlying the pathogenesis of this disorder. Furthermore, it has been suggested that the cytopenias associated with T-LGL leukemia would be the result of highly specific recognition and killing of individual hematopoietic cell lineages by T-LGL clones.

In the present study, we identified and characterized TCR clonotypes in a group of CD8^+^/TCRαβ^+^ T-LGL leukemia patients. We could not detect specific predominant Vβ family usage in our CD8^+^/TCRαβ^+^ T-LGL cohort. Immunophenotypical analysis of TCR Vα expression has so far only been explored in a minority of TCRαβ^+^ T-LGL leukemia cases. Likewise, complete sequencing of TCRA gene rearrangements has only been performed in few (*n*=5) T-LGL leukemia patients, thus far not showing any signs of common Vα or Jα gene usage.^42^ In our cohort of 22 patients, we could not detect preferential Vα or Jα gene usage; moreover, TCRA CDR3 sequence analysis did not show a common amino-acid motif between the various patients, which is thus in line with the TCRB results.

*In silico* analysis using a recent purpose-built bioinformatics method did not identify common TCRB CDR3 amino-acid sequences in a large cohort of CD8^+^ T-LGL leukemia patients. This is in strong contrast to monoclonal CD4^+^/Vβ13.1^+^ T-LGL proliferations, in which virtually all published TCRB CDR3 sequences can be assigned to one cluster with unique characteristics. Our current results therefore underline the distinct pathogenesis between the CD4^+^ and CD8^+^ T-LGL disease entities.

On the basis of our findings, we did not find straightforward evidence for common (super)antigen involvement in the pathogenesis of CD8^+^/TCRαβ^+^ T-LGL leukemia, since no immunodominant clones with identical or highly similar TCRA and TCRB CDR3 amino-acid sequences could be identified in a majority of patients. In the study of Wlodarski *et al.*^29^ identical expanded clonotypes were found in only 2 out of 56 patients. However, clonotypes specific for malignant clones were not encountered to a great extent in 172 clones from healthy individuals. We did not encounter the 108 clonotypes of the 82 evaluated leukemic LGL cases in CD8^+^ T cells of healthy individuals (data not shown), although high-throughput analysis of the TCRB repertoire of CD8^+^ T cells would be needed to draw firm conclusions. Recently, deep sequencing of the T-cell repertoire in healthy controls and CD8^+^ T-LGL leukemia has been performed and confirms that T-LGL clonotypes are not present in the general public and are therefore private to the disease.^43^

The heterogeneity of TCR clonotypes in clonal CD8^+^/TCRαβ^+^ T-LGL proliferations is partially explained by the presence of biclonal LGL proliferations and clonal switching. Both have been described in T-LGL leukemia and are suggestive of pervasive antigenic drive.^22^ In addition, Clemente *et al.*^43^ recently demonstrated that individual T-LGL clones were present at basal levels in almost all other T-LGL leukemia cases. This suggests the presence of an as yet undefined mechanism whereby certain clonotypes may predispose an individual toward the extreme monoclonal expansions commonly found in T-LGL leukemia.^43^ Another explanation for the seemingly low level of similarity between the dominant clonotypes in our study might be the large variability in HLA genotype in our patient series. Peptide binding is affected through the physical amino-acid properties and the tertiary CDR3 structure. Therefore, the linear amino-acid homology comparisons might not be the most appropriate method to identify common motifs in TCR molecules.

Altogether this may suggest that CD8^+^/TCRαβ^+^ T-LGL clones could have evolved in a stepwise manner from an initial polyclonal/oligoclonal immune response directed against multiple (auto)antigenic targets. Following an exaggerated immune response, secondary molecular events would then lead to global deregulation of cell proliferation and survival of one or a few clonotypes. Among the survival signalling pathways, the Janus kinase/signal transducer and activator of transcription (JAK/STAT) pathway has been associated with LGL transformation.^26^ Most recently, the role of STAT family genes (STAT3 and STAT5b) in the pathogenesis of T-LGL leukemia has been emphasized.^44^ Activating somatic mutations in STAT3 were found in up to 40% of T-LGL leukemia patients and it has been suggested that mutational analysis of STAT3 might distinguish true T-LGL leukemia cases from clonally skewed reactive processes.^11, 45, 46^

In summary, no clear indications for common TCRA or TCRB CDR3 motifs were found in our CD8^+^/TCRαβ^+^ T-LGL cohort. When clonotypes of our 26 patients were cross-referenced against the previously reported clonotypic database of 56 T-LGL leukemia patients, we could only identify homologous clonotypes between a limited number of patients. This is in contrast to the shared clonotypes as seen in CD4^+^/TCRαβ^+^ and TCRγδ^+^ T-LGL leukemia and might point to a more random clonal selection in TCRαβ^+^ T-LGL leukemia. The heterogeneity of clonal CD8^+^/TCRαβ^+^ T-LGL proliferations might in fact suggest that multiple pathogens or autoantigens are involved. Additional studies taking into account the triad of HLA genotype, peptide-groove binding and TCR specificity are needed to precisely define the impact of (auto)antigen stimulation in the pathogenesis of CD8^+^/TCRαβ^+^ T-LGL leukemia.

## Figures and Tables

**Table 1:** Characteristics, clinical presentation and immunophenotype of 26 patients diagnosed with CD8^+^/TCRαβ^+^ T-LGL leukemia

*Case no.*	*Sample*	*Age, sex*	*Immunophenotype*	*Main clinical presentations*^a^	*Associated disease*	*LGL clone size ( × 10*^*9*^*/l)*	*Therapy*
1	86-041	58, F	CD2/3/8/5/7	Anemia	PRCA	21.6	RBC transfusion, chlorambucil
2	96-013	58, F	CD2/3/8/16/56/57	Anemia/neutropenia	—	13.0	Unknown
3	96-043	73, F	CD2/3/8/5/7/16/56/57	Neutropenia/thrombocytopenia	Oligo arthritis	9.9	MTX
4	92-050	50, M	CD2/3/8/5/7/16neg/56neg/57/HLA DR	Neutropenia	RA (RF+), DLBCL	1.9	None
5	98-126	73, M	CD2/3/8/5/7/16/56/57/HLA DR	Anemia/thrombocytopenia	HCC	1.0	Chlorambucil
6	92-024	55, F	CD2/3/8/2/5/7/16/56neg/57/HLA DR	Neutropenia/anemia	AIHA, ITP, bronchus carcinoma	7.4	CSA
7	93-027	53, M	CD2/3/8/5/7/16neg/56neg/57/HLA DR	Anemia	AIHA	2.0	Unknown
8	96-067	39, F	CD2/3/8/5/16/57	Neutropenia	—	5.9	None
9	97-064	71, M	CD2/3/8/16neg/56neg/57/HLA DR neg	Neutropenia	—	1.2	Unknown
10	99-100	49, M	CD2/3/8/5/7/16/56neg/57/HLA DR neg	Anemia	—	5.5	Unknown
11	98-194	38, F	CD2/3/8/5/7/16/56/57neg/HLA DR	Neutropenia	—	6.6	None
12	05-060	70, M	CD2/3/8/5/7/16neg/56neg/57/HLA DR	Neutropenia/thrombocytopenia	—	3.1	Unknown
13	05-100	31, F	CD2/3/8/5/7/57	Neutropenia/anemia	—	2.0	Corticosteroids, CSA
14	05-191	73, M	CD2/3/8/5/7/16neg/56neg/57/HLA DR	Neutropenia/recurrent infections	Chronic NK-LGL leukemia	4.4	MTX
15	03-030	41, F	CD2/3/8/5/7neg/57/HLA DR	Anemia/B symptoms	PRCA	2.5	CSA
16	03-086	39, F	CD2/3/8/5/7/16/56neg/57	Fatigue	M. Hodgkin	2.0	None
17	00-113	72, M	CD2/3/8/5neg/7/16neg/56neg/57/HLA DRneg	Neutropenia/ B symptoms	—	3.5	None
18	93-054	62, M	CD2/3/8/7/16neg/57	Anemia/splenomegaly/B symptoms	Chronic osteomyelitis, melanoma	4.4	Chlorambucil, RBC transfusion
19	02-047	76, F	CD2/3/8/5/7/57/HLA DR	B symptoms	—	12.0	None
20	91-004	86, F	CD2/3/8/7/16neg/56neg	Neutropenia/anemia/ lymphadenopathy	—	3.8	None
21	05-281	59, M	CD2/3/8/5/7neg/16neg/56/57/HLA DRneg	B symptoms	—	2.7	None
22	06-026	67, M	CD2neg/3/8/5/7/16/56neg/57/HLA DRneg	Neutropenia/B symptoms/ thrombocytopenia	(Oligo) RA	2.0	HCQ
23	06-038	69, M	CD2/3/8/5neg/7/16neg/56neg/57/HLA DR	Neutropenia	RA	3.2	MTX
24	06-127	35, F	CD2/3/8/5/7/16/56neg/57/HLA DR	Neutropenia	—	3.7	None
25	06-131	61, F	CD2/3/8/5/7/16neg/56/57/HLA DR	Recurrent infections	—	1.2	None
26	06-246	47, F	CD2/3/8/5/7/16/56/57/HLA DR	Neutropenia/splenomegaly	—	10.2	CSA

Abbreviations: AIHA, autoimmune hemolytic anemia; CSA, cyclosporin A; DLBCL, diffuse large B-cell lymphoma; HCC, hepatocellular carcinoma; HCQ, hydroxychloroquine; ITP, idiopathic thrombocytopenic purpura; MTX, methotrexate; PRCA, pure red cell aplasia; RA, rheumatoid arthritis; RBC, red blood cell; RF, rheumatoid factor; TCR, T-cell receptor; T-LGL, T-cell large granular lymphocyte.

a Neutropenia was defined as absolute neutrophil count (ANC) <1.5 × 10^9^ neutrophils/l; anemia was defined as hemoglobin level <10 g/dl; thrombocytopenia was defined as platelet count <150 × 10^9^/l.

**Table 2:** HLA genotype and Vα/Vβ usage in CD8^+^/TCRαβ^+^ T-LGL leukemia patients

*Case no.*	*Sample no.*	*LGL expansion*	*Expanded TCR Vβ family*^a^	*Expanded TCR Vα family*^b^	*HLA-A*	*HLA-B*	*HLA-C*
1	86-041	Oligoclonal	Vβ1	Vα19	02, 11	35, 50	04, 06
2	96-013	Oligoclonal	Vβ12	Vα19	02, 68	40, 44	03, 07
3	96-043	Monoclonal	Vβ22	Vα29	01, 25	07, 08	03, 07
4	92-050	Oligoclonal	Vβ23	Vα17	02, 02	13, 15	03, 12
5	98-126	Oligoclonal	Vβ2	Vα19	ND	ND	ND
6	92-024	Monoclonal	Vβ7.2	Vα12.2	01, 24	08, 40	03, 07
7	93-027	Oligoclonal	Vβ8.1/8.2	Vα6	02, 02	40, 40	03, 03
8	96-067	Oligoclonal	No reactivity (Vβ6 in PCR)	Vα26	02, 31	27, 58	02, 07
9	97-064	Oligoclonal	Vβ13	Vα35	24, 29	45, 49	06, 07
10	99-100	Monoclonal	Vβ17	Vα23	01, 02	08, 41	07, 17
11	98-194	Biclonal	No reactivity (Vβ6/Vβ12 in PCR)	Vα6/Vα21	02, 03	07, 27	01, 07
12	05-060	Monoclonal	Vβ17	Vα30/Vα26	03, 32	41, 55	03, 17
13	05-100	Monoclonal	Vβ16	Vα12.2	02, 03	18, 51	05, 14
14	05-191	Monoclonal	Vβ5	Vα1	02, 02	15, 35	03, 04
15	03-030	Biclonal	No reactivity (Vβ6/Vβ16 in PCR)	ND	02, 02	08, 08	07, 07
16	03-086	Monoclonal	Vβ14	Vα3	02, 03	08, 35	04, 07
17	00-113	Monoclonal	Vβ8.2	Vα8	01, 26	38, 44	05, 12
18	93-054	Oligoclonal	Vβ12/Vβ15	Vα12.3	01, 02	15, 57	03, 06
19	02-047	Monoclonal	Vβ7.2	Vα8	ND	ND	ND
20	91-004	Biclonal	Vβ5/Vβ6	Vα41	ND	ND	ND
21	05-281	Monoclonal	Vβ2	Vα1.2	ND	ND	ND
22	06-026	Monoclonal	No reactivity (Vβ24 in PCR)	Vα29	ND	ND	ND
23	06-038	Monoclonal	Vβ2	Vα12.1	01, 24	08, 18	05, 07
24	06-127	Monoclonal	Vβ13	ND	01, 11	27, 35	02, 04
25	06-131	Monoclonal	Vβ12	ND	ND	ND	ND
26	06-246	Monoclonal	Vβ5.1	ND	ND	ND	ND

Abbreviations: ND, not done; TCR, T-cell receptor; T-LGL, T-cell large granular lymphocyte.

a Dominant TCR Vβ family usage defined by immunophenotyping and molecular analysis.

b Dominant TCR Vα family usage defined by molecular analysis.

**Table 3:** Amino-acid sequences of CDR3 motifs of in-frame TCRB rearranged alleles in patients with CD8^+^/TCRαβ^+^ T-LGL leukemia

*Case no.*	*Sample no.*	*Rearrangement*	*V*							*N-(D)-N*											*J*								
1	86-041	Vβ1-Jβ2.1	C	A	S	S				L	S	G	R	A	L										N	E	Q	F	F
2	96-013	Vβ12.1-Jβ2.2	C	A	I	S	E			G	S	G	P												G	E	L	F	F
3	96-043	Vβ22-Jβ2.6	C	A	S					G	G	D	R	G	T								G	A	N	V	L	T	F
4	92-050	Vβ23-Jβ1.5	C	A	S	S	L			G	G	R	Y										S	N	Q	P	Q	H	F
5	98-126	Vβ2-Jβ1.3	C	S	A					S	L	G	G	R	P	T	I	A						G	N	T	I	Y	F
6	92-024	Vβ7.2-Jβ1.5	C	A	S	S	Q			D	V	R	P	P	P	E	D	R	P	Y			S	N	Q	P	Q	H	F
7	93-027	Vβ8.2-Jβ2.5	C	A	S	S	L			G	T	G	G	M											E	T	Q	Y	F
8	96-067	Vβ6.2-Jβ2.1	C	A	S	S				L	A	H											S	Y	N	E	Q	F	F
9	97-064	Vβ13.3-Jβ2.7	C							G	S	L	G	Q	G	A	W								Y	E	Q	Y	F
10	99-100	Vβ17-Jβ2.7	C	A	S					S	P	E	S	L	F									S	Y	E	Q	Y	F
11	98-194	Vβ6.4-Jβ1.1	C	A						R	S	F	S	P	S	L	D	T			S	S	L	F	V	E	A	F	F
		Vβ12.3-Jβ2.3	C	A	S	S				C	Y	Q	P	G	L	D					L	P	R	A	D	T	Q	Y	F
12	05-060	Vβ17-Jβ2.7	C	A	S	S				I	F	R	G	N												E	Q	Y	F
13	05-100	Vβ16-Jβ2.5	C	A	S	S				P	V	G	A	Y	P	K									E	T	Q	Y	F
14	05-191	Vβ5.1-Jβ1.4	C	A	S	S	L			A	K	G	K	G	A								T	N	E	K	L	F	F
15	03-030	ND																											
16	03-086	Vβ14-Jβ2.1	C	A	S					N	N	R	G	S										Y	N	E	Q	F	F
17	00-113	Vβ8.2-Jβ1.5	C	A	S	S				W	G	G												N	Q	P	Q	H	F
18	93-054	Vβ12.1-Jβ2.3	C	A	I	S				G	R	L	A	G	G	R	T						S	T	D	T	Q	Y	F
19	02-047	Vβ7.2-Jβ2.2	C	A	S					G	G	V	G	G											G	E	L	F	F
20	91-004	Vβ5.6-Jβ2.1	C	A	S	S				L	D	N												Y	N	E	Q	F	F
		Vβ6.5-Jβ1.1	C	A	S	S				F	S	P	Y	T	R	P										E	A	F	F
21	05-281	Vβ2-Jβ1.4	C	A						V	P	T	G	R										N	E	K	L	F	F
22	06-026	Vβ24-Jβ2.7	C	A	T	S				R	D	L	L	T											Y	E	Q	Y	F
23	06-038	Vβ2-Jβ2.5	C	S	A					R	N	G	P	N	Y									Q	E	T	Q	Y	F
24	06-127	Vβ13.6-Jβ1.5	C	A	S	S				Y	G	S	P	L	D	I	D	S	A	I	S					P	Q	H	F
25	06-131	Vβ12.2-Jβ1.2	C	A	S	S				P	K	G													Y	G	Y	T	F
26	06-246	Vβ5.1-Jβ1.2	C	A	S	S	L			G	S	G													Y	G	Y	T	F

Abbreviations: CDR3, complementarity determining region 3; ND, not done; TCRB, T-cell receptor β T-LGL, T-cell large granular lymphocyte.

A L I V G(P): neutral side chain; D E: acidic side chain; S T: aliphatic side chain; N Q: amide side chain; K R H: basic side chain; M: sulfur-containing side chain; F Y W: cyclic side chain.

**Table 4:** Amino-acid sequences of CDR3 motifs of in-frame TCRA rearranged alleles in patients with CD8^+^/TCRαβ^+^ T-LGL leukaemia

*Case no.*	*Sample no.*	*Rearrangement*	*V*						*N-(D)-N*							*J*										
1	86-041	Vα19-Jα49	C	A	L	S	E		S	G											G	N	Q	F	Y	F
2	96-013	Vα19-Jα26	C	A	L	S	E		G	S	R	F								Y	G	Q	N	F	V	F
3	96-043	Vα29-Jα52	C						G	R	V										A	G	G	T	S	F
4	92-050	Vα17-Jα20	C	A					T	L	S								S	N	D	Y	K	L	S	F
5	98-126	Vα19-Jα37	C	A	L	S	E		A	E	G	S							S	N	T	G	K	L	I	F
6	92-024	Vα12.2-Jα53	C	A					V	T								G	G	S	N	Y	K	L	T	F
7	93-027	Vα6-Jα21	C						V	G											F	N	K	F	Y	F
8	96-067	Vα26.2-Jα20	C	I					P	S	P	S								N	D	Y	K	L	S	F
9	97-064	Vα35-Jα49	C	A					G	F										T	G	N	Q	F	Y	F
10	99-100	Vα23-Jα52	C	A					A	P	V						G	G	T	S	Y	G	K	L	T	F
11	98-194	Vα8.6-Jα56	C	A	V	S			L									T	G	A	N	S	K	L	T	F
		Vα21-Jα57	C	A	V				K										G	G	S	E	K	L	V	F
12	05-060	Vα26.2-Jα24	C	I	L	R	D		V	E	S	W	G	K	F	Q										F
		Vα30-Jα44	C	G					T	P	G	N							G	T	A	S	K	L	T	F
13	05-100	Vα12.2-Jα23	C	A	V															Q	G	G	K	L	I	F
14	05-191	Vα1.1-Jα10	C	A					V	D	P	G	L	A	A					G	G	N	K	L	T	F
15	03-030	ND																								
16	03-086	Vα3-Jα36	C	A					S	D								Q	T	G	A	N	N	L	F	F
17	00-113	Vα8.1-Jα28	C	A	V				M							Y	S	G	A	G	S	Y	Q	L	T	F
18	93-054	Vα12.3-Jα9	C	A	M	S			A	V	M	R									G	F	K	T	I	F
19	02-047	Vα8.1-Jα39	C	A	V				M	S	D									A	G	N	M	L	T	F
20	91-004	Vα41-Jα48	C	A	V				N																	F
21	05-281	Vα1-2-Jα33	C	A					T	L									D	S	N	Y	Q	L	I	W
22	06-026	Vα29-Jα45	C	A	A				K	G	F												G	L	T	F
23	06-038	Vα12.1-Jα34	C	V	V				K									S	Y	N	T	D	K	L	I	F
24	06-127	ND																								
25	06-131	ND																								
26	06-246	ND																								

Abbreviations: CDR3, complementarity determining region 3; ND, not done; TCRA, T-cell receptor α T-LGL, T-cell large granular lymphocyte.

A L I V G (P): neutral side chain; D E: acidic side chain; S T: aliphatic side chain; N Q: amide side chain; K R H: basic side chain; C M: sulfur-containing side chain; F Y W: cyclic side chain.

## References

[bib1] Langerak AW Sandberg Y van Dongen JJ Spectrum of T-large granular lymphocyte lymphoproliferations: ranging from expanded activated effector T cells to T-cell leukaemia Br J Haematol 2003 123 561 562 14617025 10.1046/j.1365-2141.2003.04647.x

[bib2] Loughran TP Jr Clonal diseases of large granular lymphocytes Blood 1993 82 1 14 8324214

[bib3] Sokol L Loughran TP Jr Large granular lymphocyte leukemia Oncologist 2006 11 263 273 16549811 10.1634/theoncologist.11-3-263

[bib4] Dhodapkar MV Li CY Lust JA Tefferi A Phyliky RL Clinical spectrum of clonal proliferations of T-large granular lymphocytes: a T-cell clonopathy of undetermined significance Blood 1994 84 1620 1627 8068951

[bib5] Lamy T Loughran TP Jr Current concepts: large granular lymphocyte leukemia Blood Rev 1999 13 230 240 10741898 10.1054/blre.1999.0118

[bib6] Lamy T Loughran TP Jr Clinical features of large granular lymphocyte leukemia Semin Hematol 2003 40 185 195 12876667 10.1016/s0037-1963(03)00133-1

[bib7] Rose MG Berliner N T-cell large granular lymphocyte leukemia and related disorders Oncologist 2004 9 247 258 15169980 10.1634/theoncologist.9-3-247

[bib8] Bareau B Rey J Hamidou M Donadieu J Morcet J Reman O Analysis of a French cohort of patients with large granular lymphocyte leukemia: a report on 229 cases Haematologica 2010 95 1534 1541 20378561 10.3324/haematol.2009.018481 PMC2930955

[bib9] Lamy T Loughran TP Jr How I treat LGL leukemia Blood 2011 117 2764 2774 21190991 10.1182/blood-2010-07-296962 PMC3062292

[bib10] Semenzato G Zambello R Starkebaum G Oshimi K Loughran TP Jr The lymphoproliferative disease of granular lymphocytes: updated criteria for diagnosis Blood 1997 89 256 260 8978299

[bib11] Jerez A Clemente MJ Makishima H Koskela H Leblanc F Ng KP STAT3 mutations unify the pathogenesis of chronic lymphoproliferative disorders of NK cells and T cell large granular lymphocyte leukemia Blood 2012 120 3048 3057 22859607 10.1182/blood-2012-06-435297 PMC3471515

[bib12] Dearden C Large granular lymphocytic leukaemia pathogenesis and management Br J Haematol 2011 152 273 283 21158751 10.1111/j.1365-2141.2010.08494.x

[bib13] Alekshun TJ Tao J Sokol L Aggressive T-cell large granular lymphocyte leukemia: a case report and review of the literature Am J Hematol 2007 82 481 485 17205534 10.1002/ajh.20853

[bib14] Gentile TC Uner AH Hutchison RE Wright J Ben-Ezra J Russell EC CD3+, CD56+ aggressive variant of large granular lymphocyte leukemia Blood 1994 84 2315 2321 7522625

[bib15] Lima M Almeida J Dos Anjos Teixeira M Alguero Md Mdel C Santos AH Balanzategui A TCRalphabeta+/CD4+ large granular lymphocytosis: a new clonal T-cell lymphoproliferative disorder Am J Pathol 2003 163 763 771 12875995 10.1016/s0002-9440(10)63703-0 PMC1868208

[bib16] Sandberg Y Almeida J Gonzalez M Lima M Barcena P Szczepanski T TCRgammadelta+ large granular lymphocyte leukemias reflect the spectrum of normal antigen-selected TCRgammadelta+ T-cells Leukemia 2006 20 505 513 16437145 10.1038/sj.leu.2404112

[bib17] Sabnani I Tsang P Are clonal T-cell large granular lymphocytes to blame for unexplained haematological abnormalities Br J Haematol 2007 136 30 37 17092307 10.1111/j.1365-2141.2006.06374.x

[bib18] O'Keefe CL Plasilova M Wlodarski M Risitano AM Rodriguez AR Howe E Molecular analysis of TCR clonotypes in LGL: a clonal model for polyclonal responses J Immunol 2004 172 1960 1969 14734782 10.4049/jimmunol.172.3.1960

[bib19] Sokol L Agrawal D Loughran TP Jr Characterization of HTLV envelope seroreactivity in large granular lymphocyte leukemia Leuk Res 2005 29 381 387 15725471 10.1016/j.leukres.2004.08.010

[bib20] Starkebaum G Loughran TP Jr Kalyanaraman VS Kadin ME Kidd PG Singer JW Serum reactivity to human T-cell leukaemia/lymphoma virus type I proteins in patients with large granular lymphocytic leukaemia Lancet 1987 1 596 599 2881134 10.1016/s0140-6736(87)90236-4

[bib21] Zambello R Trentin L Facco M Cerutti A Sancetta R Milani A Analysis of the T cell receptor in the lymphoproliferative disease of granular lymphocytes: superantigen activation of clonal CD3+ granular lymphocytes Cancer Res 1995 55 6140 6145 8521405

[bib22] Clemente MJ Wlodarski MW Makishima H Viny AD Bretschneider I Shaik M Clonal drift demonstrates unexpected dynamics of the T-cell repertoire in T-large granular lymphocyte leukemia Blood 2011 118 4384 4393 21865345 10.1182/blood-2011-02-338517 PMC3204910

[bib23] Lamy T Liu JH Landowski TH Dalton WS Loughran TP Jr Dysregulation of CD95/CD95 ligand-apoptotic pathway in CD3(+) large granular lymphocyte leukemia Blood 1998 92 4771 4777 9845544

[bib24] Liu JH Wei S Lamy T Li Y Epling-Burnette PK Djeu JY Blockade of Fas-dependent apoptosis by soluble Fas in LGL leukemia Blood 2002 100 1449 1453 12149230

[bib25] Yang J Epling-Burnette PK Painter JS Zou J Bai F Wei S Antigen activation and impaired Fas-induced death-inducing signaling complex formation in T-large-granular lymphocyte leukemia Blood 2008 111 1610 1616 17993614 10.1182/blood-2007-06-093823 PMC2214759

[bib26] Teramo A Gattazzo C Passeri F Lico A Tasca G Cabrelle A Intrinsic and extrinsic mechanisms contribute to maintain the JAK/STAT pathway aberrantly activated in T-type large granular lymphocyte leukemia Blood 2013 121 (3843-3854 S1 10.1182/blood-2012-07-441378 23515927

[bib27] Borg NA Ely LK Beddoe T Macdonald WA Reid HH Clements CS The CDR3 regions of an immunodominant T cell receptor dictate the 'energetic landscape' of peptide-MHC recognition Nat Immunol 2005 6 171 180 15640805 10.1038/ni1155

[bib28] Garrido P Ruiz-Cabello F Barcena P Sandberg Y Canton J Lima M Monoclonal TCR-Vbeta13.1+/CD4+/NKa+/CD8-/+dim T-LGL lymphocytosis: evidence for an antigen-driven chronic T-cell stimulation origin Blood 2007 109 4890 4898 17303697 10.1182/blood-2006-05-022277

[bib29] Wlodarski MW O'Keefe C Howe EC Risitano AM Rodriguez A Warshawsky I Pathologic clonal cytotoxic T-cell responses: nonrandom nature of the T-cell-receptor restriction in large granular lymphocyte leukemia Blood 2005 106 2769 2780 15914562 10.1182/blood-2004-10-4045

[bib30] Kjer-Nielsen L Clements CS Purcell AW Brooks AG Whisstock JC Burrows SR A structural basis for the selection of dominant alphabeta T cell receptors in antiviral immunity Immunity 2003 18 53 64 12530975 10.1016/s1074-7613(02)00513-7

[bib31] Herling M Khoury JD Washington LT Duvic M Keating MJ Jones D A systematic approach to diagnosis of mature T-cell leukemias reveals heterogeneity among WHO categories Blood 2004 104 328 335 15044256 10.1182/blood-2004-01-0002

[bib32] van den Beemd R Boor PP van Lochem EG Hop WC Langerak AW Wolvers-Tettero IL Flow cytometric analysis of the Vbeta repertoire in healthy controls Cytometry 2000 40 336 345 10918284 10.1002/1097-0320(20000801)40:4<336::aid-cyto9>3.0.co;2-0

[bib33] Langerak AW van Den Beemd R Wolvers-Tettero IL Boor PP van Lochem EG Hooijkaas H Molecular and flow cytometric analysis of the Vbeta repertoire for clonality assessment in mature TCRalphabeta T-cell proliferations Blood 2001 98 165 173 11418476 10.1182/blood.v98.1.165

[bib34] van Dongen JJ Wolvers-Tettero IL Analysis of immunoglobulin and T cell receptor genes. Part I: Basic and technical aspects Clin Chim Acta 1991 198 1 91 1863985 10.1016/0009-8981(91)90246-9

[bib35] Beillard E Pallisgaard N van der Velden VH Bi W Dee R van der Schoot E Evaluation of candidate control genes for diagnosis and residual disease detection in leukemic patients using 'real-time' quantitative reverse-transcriptase polymerase chain reaction (RQ-PCR) - a Europe against cancer program Leukemia 2003 17 2474 2486 14562124 10.1038/sj.leu.2403136

[bib36] van Dongen JJ Langerak AW Bruggemann M Evans PA Hummel M Lavender FL Design and standardization of PCR primers and protocols for detection of clonal immunoglobulin and T-cell receptor gene recombinations in suspect lymphoproliferations: report of the BIOMED-2 Concerted Action BMH4-CT98-3936 Leukemia 2003 17 2257 2317 14671650 10.1038/sj.leu.2403202

[bib37] Langerak AW Szczepanski T van der Burg M Wolvers-Tettero IL van Dongen JJ Heteroduplex PCR analysis of rearranged T cell receptor genes for clonality assessment in suspect T cell proliferations Leukemia 1997 11 2192 2199 9447840 10.1038/sj.leu.2400887

[bib38] Breit TM Van Dongen JJ Unravelling human T-cell receptor junctional region sequences Thymus 1994 22 177 199 7940645

[bib39] Darzentas N Circoletto: visualizing sequence similarity with Circos Bioinformatics 2010 26 2620 2621 20736339 10.1093/bioinformatics/btq484

[bib40] Darzentas N Hadzidimitriou A Murray F Hatzi K Josefsson P Laoutaris N A different ontogenesis for chronic lymphocytic leukemia cases carrying stereotyped antigen receptors: molecular and computational evidence Leukemia 2010 24 125 132 19759557 10.1038/leu.2009.186

[bib41] Freeman JD Warren RL Webb JR Nelson BH Holt RA Profiling the T-cell receptor beta-chain repertoire by massively parallel sequencing Genome Res 2009 19 1817 1824 19541912 10.1101/gr.092924.109 PMC2765271

[bib42] Kasten-Sportes C Zaknoen S Steis RG Chan WC Winton EF Waldmann TA T-cell receptor gene rearrangement in T-cell large granular leukocyte leukemia: preferential V alpha but diverse J alpha usage in one of five patients Blood 1994 83 767 775 8298138

[bib43] Clemente MJ Przychodzen B Jerez A Dienes BE Afable MG Husseinzadeh H Deep sequencing of the T cell receptor repertoire in CD8+ T-large granular lymphocyte leukemia identifies signature landscapes Blood 2013 122 4077 4085 24149287 10.1182/blood-2013-05-506386 PMC3862272

[bib44] Rajala HL Eldfors S Kuusanmaki H van Adrichem AJ Olson T Lagstrom S Discovery of somatic STAT5b mutations in large granular lymphocytic leukemia Blood 2013 121 4541 4550 23596048 10.1182/blood-2012-12-474577 PMC3668487

[bib45] Koskela HL Eldfors S Ellonen P van Adrichem AJ Kuusanmaki H Andersson EI Somatic STAT3 mutations in large granular lymphocytic leukemia N Engl J Med 2012 366 1905 1913 22591296 10.1056/NEJMoa1114885 PMC3693860

[bib46] Ohgami RS Ma L Merker JD Martinez B Zehnder JL Arber DA STAT3 mutations are frequent in CD30+ T-cell lymphomas and T-cell large granular lymphocytic leukemia Leukemia 2013 27 2244 2247 23563237 10.1038/leu.2013.104

[bib47] Krzywinski M Schein J Birol I Connors J Gascoyne R Horsman D Circos: an information aesthetic for comparative genomics Genome Res 2009 19 1639 1645 19541911 10.1101/gr.092759.109 PMC2752132

