# Supplementary material for: Lack of common TCRA and TCRB clonotypes in CD8+/TCRαβ+ T-cell large granular lymphocyte leukemia: a review on the role of antigenic selection in the immunopathogenesis of CD8+ T-LGL
Source: Blood Cancer J. 2014 Jan 10;4(1):e172–. doi: 10.1038/bcj.2013.70 (PMC3913939; doi:10.1038/bcj.2013.70)
Supplement: Supplementary Table 1 [file bcj201370x1.doc]

# Supplementary Table 1. Composition of TCRA multiplex PCR tubes

| **Tube A** |  | **Size of PCR product** |  |  |  |
| --- | --- | --- | --- | --- | --- |
| Vα1-1 | GAGCCATTGTCCAGATAAACTG | 232 | 30 | Cα | GGTACACGGCAGGGTCAG |
| Vα8-4 | GGAGCCCTGGTTCTGCTG | 239 |  |  |  |
| Vα8-7 | AAGAAGCCCCTCTGGAACTG | 247 |  |  |  |
| Vα12-1 | CCAGAGGGAGCCACTGTC | 235 |  |  |  |
| Vα12-2 | GAGCCATTGCCTCTCTCAACT | 231 |  |  |  |
| Vα12-3 | GGGAGCCATTGTTTCTCTCA | 233 |  |  |  |
| Vα30 | GGGGAAGATGCTGTCATCAA | 231 |  |  |  |
| Vα35 | GGAGAAGATGTCTCCATGAACT | 231 |  |  |  |
| Vα39 | CATGCAGGAGGGAAAAAACT | 242 |  |  |  |
| Vα40 | GGAGGGAGCATCTGTGACT | 225 |  |  |  |
| Vα41 | GCCCAGGAAGGAGAATTTATCA | 234 |  |  |  |
|  |  |  |  |  |  |
| **Tube B** |  |  |  |  |  |
| Vα3 | GTTGCTGAAGGGAATCCTCT | 250 | 30 | Cα | GGTACACGGCAGGGTCAG |
| Vα4 | AGAAGTGAACATAACCTGTAGCCA | 240 |  |  |  |
| Vα9-2 | AAACTGCACGTACACAGCCA | 219 |  |  |  |
| Vα13-1 | GAGACAGCGCTGTTATCAAGTG | 234 |  |  |  |
| Vα16 | TTTAAAGGGGCCCCAGTG | 233 |  |  |  |
| Vα17 | AGGAGGGTGAAAATGCCA | 237 |  |  |  |
| Vα18 | CCAGTTACCCTCCCTGAGAG | 251 |  |  |  |
| Vα31 | GTGAGACCGTGAAACTGGACT | 243 |  |  |  |
| Vα33 | AGGCAGAAAGGAGTAGCTGTGA | 243 |  |  |  |
| Vα38-2 | TCTGTGCAGGAGGCAGAGA | 256 |  |  |  |
|  |  |  |  |  |  |
| **Tube C** |  |  |  |  |  |
| Vα5 | ACAGCTCCGTTATAAACTGCAC | 231 | 30 | Cα | GGTACACGGCAGGGTCAG |
| Vα8-1 | GCCTCACTGGAGTTGGGAT | 236 |  |  |  |
| Vα22 | GGAGGGAGCCAATTCCAC | 230 |  |  |  |
| Vα24 | GGGAGACAGCACCAATTTCA | 233 |  |  |  |
| Vα26-2 | GAGCCTGTTCACTTGCCTTG | 231 |  |  |  |
| Vα27 | GGGAGAAAATCTCACTGTGTACT | 230 |  |  |  |
| Vα29 | GCGTCCAGGAAGGAAGAATTT | 246 |  |  |  |
| Vα32 | GAAATGGCCGTTATTAATGACA | 235 |  |  |  |
| Vα36 | GGGAGACACCGTAACTCTCAAT | 233 |  |  |  |
| Vα37 | GGAAGGTGACAGCGTCACA | 238 |  |  |  |
| Vα38-1 | GCAGGAGGCAGAGACTGTG | 252 |  |  |  |
|  |  |  |  |  |  |
| **Tube D** |  |  |  |  |  |
| Vα2 | GGAGCTGTGGTGGAAATCT | 221 | 30 | Cα | GGTACACGGCAGGGTCAG |
| Vα6 | ACATTCAGGAGGGTAAAACGG | 246 |  |  |  |
| Vα7 | CGTTGCCTCCATGAGCTG | 224 |  |  |  |
| Vα8-3 | GGAGCCTCACTGGAGTTGAGA | 239 |  |  |  |
| Vα8-5 | GAAGGAGCCTCACTGGAGTT | 315 |  |  |  |
| Vα8-6 | AAGCCCCTGTGGAGCTG | 238 |  |  |  |
| Vα19 | TGGTGGAGAAGGAGGATGTG | 253 |  |  |  |
| Vα20 | CAGGAGGGAGAGAGTAGCAGTC | 235 |  |  |  |
| Vα23 | CCAGAAAGGAGGGATTTCAATTA | 242 |  |  |  |
| Vα26-1 | GCTGCAAACCTGCCTTGTAA | 229 |  |  |  |
| Vα28 | GAAGAAATTCATTCCTGGTGTG | 223 |  |  |  |
|  |  |  |  |  |  |
| **Tube E** |  |  |  |  |  |
| Vα1-2 | GTGCCATTGTCCAGATCAACT | -232 | 30 | Cα | GGTACACGGCAGGGTCAG |
| Vα8-2 | AACCCCGGTGCTGCTG | -237 |  |  |  |
| Vα9-1 | TTCCCTGATTGTGAACTGCTC | -231 |  |  |  |
| Vα10 | GGAAAGAACTGCACTCTTCAA | -235 |  |  |  |
| Vα11 | GGAATGCATGCCGTTCTTAAT | -231 |  |  |  |
| Vα13-2 | CCAGGAGGGTGACAACTCTATT | -242 |  |  |  |
| Vα14 | AGGAGGCTGTGACTCTGGACT | -244 |  |  |  |
| Vα15 | GGGAATGCACAACATTCTTAAT | -236 |  |  |  |
| Vα21 | AAGGAGAAAACTTGGTTCTCAACT | -236 |  |  |  |
| Vα25 | GGAGAGGACTTCACCACGTACT | -228 |  |  |  |
| Vα34 | GGGAAAGAATCTCACCATAAACT | -243 |  |  |  |
