# Supplementary figures and images for: Lack of common TCRA and TCRB clonotypes in CD8+/TCRαβ+ T-cell large granular lymphocyte leukemia: a review on the role of antigenic selection in the immunopathogenesis of CD8+ T-LGL
Source: Blood Cancer J. 2014 Jan 10;4(1):e172–. doi: 10.1038/bcj.2013.70 (PMC3913939; doi:10.1038/bcj.2013.70)

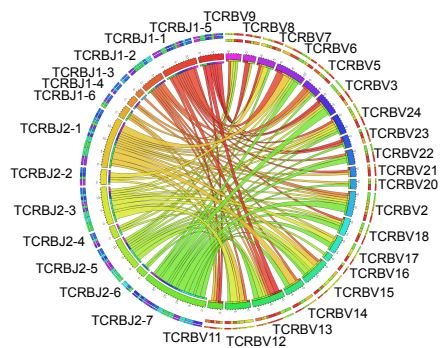

Supplement: Supplementary Figure 1 [file bcj201370x2.pdf]
