# Supplementary material for: Lack of common TCRA and TCRB clonotypes in CD8+/TCRαβ+ T-cell large granular lymphocyte leukemia: a review on the role of antigenic selection in the immunopathogenesis of CD8+ T-LGL
Source: Blood Cancer J. 2014 Jan 10;4(1):e172–. doi: 10.1038/bcj.2013.70 (PMC3913939; doi:10.1038/bcj.2013.70)
Supplement: Supplementary Figure Legend [file bcj201370x3.doc]

**Supplementary Figure 1. Frequencies of Vβ-Jβ pairing in TCRαβ^+^ T-LGL leukemia patients.** Highly diverse combinations are seen in a cohort of 82 patients with CD8^+^ T-LGL leukemia, representing 108 clonotypes.
